# Social and individual factors mediate chimpanzee vocal ontogeny

**DOI:** 10.1038/s41598-025-93207-x

**Published:** 2025-03-12

**Authors:** Adrian Soldati, Pawel Fedurek, Guillaume Dezecache, Geresomu Muhumuza, Catherine Hobaiter, Klaus Zuberbühler, Josep Call

**Affiliations:** 1 Department of Evolutionary Anthropology, University of Zürich, Zürich, Switzerland; 2 School of Psychology and Neuroscience, University of St Andrews, St Andrews, UK; 3 Department of Comparative Cognition, University of Neuchâtel, Neuchâtel, Switzerland; 4 Division of Psychology, University of Stirling, Stirling, UK; 5 UMI SOURCE, Université Paris-Saclay, IRD, UVSQ, Guyancourt, France; 6 Budongo Conservation Field Station, Masindi, Uganda

**Keywords:** Biological anthropology, Animal behaviour

## Abstract

Human language develops in social interactions. In other ape species, the role of social learning in vocal ontogeny can be typically underappreciated, mainly because it has received little empirical attention. Here, we examine the development of pant hoot vocalisations during vocal exchanges in immature wild chimpanzees (*Pan troglodytes schweinfurthii*) of the Sonso community of the Budongo Forest, Uganda. We investigated how maternal gregariousness, age, sex, and social context are associated with behavioural and vocal responses to other group members’ calls. We show that the older sons of gregarious mothers are more likely to orient their attention, respond vocally to the calls of others, and are overall more exposed to others’ calls compared to other immature individuals. This effect is strongest in the presence of adult males and when their mothers also respond vocally, suggesting that chimpanzee vocal development is enhanced by social and vocal exposure. Our findings are consistent with a more flexible and socially mediated chimpanzee vocal ontogeny than previously assumed and show some parallels with animal vocal learners and children language acquisition.

## Introduction

Language is arguably one of the key features that distinguish humans from other animal species, a fact that continues to foster scientific debate and major research efforts^1^. Language does not leave direct fossil traces, but comparative research on other species’ communicative systems can provide insights about possible precursor stages and determine whether spoken language has emerged during our evolutionary history as a continuous process^2^. The vocal communication of non-human primates, our closest living relatives, is of particular research interest and is critical to study precursors shared with other systems of communication in biologically closely related species^3^. Although this approach has turned out to be very productive, so far it is typically based on research conducted on adult individuals that are already fully-competent communicators^4^. However, considerably less researched historically and only recently experiencing more attention^5,6^, but equally important for understanding of the evolution of spoken language, is the evolution of vocal ontogeny in non-human animals, i.e., the learning processes that drive vocal communication from birth to adulthood and the cognitive apparatus responsible for it^7^.

An effective way of conceptualising vocal development is distinguishing between three processes of learning: (1) how to produce calls (production learning); (2) how to use calls appropriately (usage learning), and (3) how to form appropriate correspondence between calls and their meaning (comprehension or response learning)^8,9^. Primate vocal development has long been argued to be rather inflexible in these three domains (but see^9^), especially in terms of production learning^10^, discouraging research on vocal learning in apes^1,11^, and leading to the consensus that socially learned vocal communication has likely emerged through convergent evolution in a limited number of biologically distant species or orders (e.g., bats, cetaceans, elephants, and songbirds^12^).

In humans, social interactions and social feedback are essential for language learning^13^. Initially, human infants undergo a perceptual learning phase that precedes vocal production learning and during which they selectively attend to auditory or visual communicative signals produced by others^14^. Greater social experience and exposure to speech provide infants with more communicative opportunities, accelerating their linguistic development^15^, for example enhancing vocal production and usage learning when exposed to mothers’ responses^16^ and response learning when infants visually attend to the vocalizations of others^14^. Socially mediated vocal development has also been documented in animals, though the evidence is mostly from birds, with relatively few studies on mammals and limited evidence in primates^12,17^. A notable exception is research conducted on marmosets demonstrating that infants learn when to use and how to produce vocalisations through vocal feedback and auditory exposure from their caregivers (common marmosets, *Callithrix jacchus*^18^; pygmy marmosets, *Cebuella pigmaea*^19^). In contrast, there is a paucity of comparable evidence from great apes, despite increasing evidence for nuanced and flexible adult vocal behaviour^20^.

Here, we seek to fill in this gap by examining chimpanzee vocal ontogeny for the following reasons. First, among primates, there are fewer studies on great ape vocal ontogeny, which are particularly relevant for studies of language evolution given that they are our closest living relatives^21^. Second, several key studies on primate vocal development have been conducted in captivity or in artificial settings^22,23^, where individuals might not express the same social behaviours as in the wild as a result of altered socio-ecological conditions (e.g., restricted spaces, atypical group size and composition)^24^. Third, many developmental studies have been carried out within experimental and atypical social contexts, including social isolation (squirrel monkeys, *Saimiri sciureus*^25^), deafening (squirrel monkeys^26^), or cross-fostering (rhesus and japanese macaques, *Macaca mulatta* and *M. muscata*^23^). While these studies offered an opportunity to control some potentially influential external factors, they may have introduced limitations on the possibility of detecting social learning and are not ethically permissible on wild great apes.

Regarding vocal ontogeny, a first prerequisite concerns the ability to socially learn vocal production and usage by attending to others’ vocal behaviour, which has been demonstrated in some mammals^8,17^, including in monkeys^18^. Infant marmosets who receive more social feedback through parental responses learn to produce calls in the adult form earlier^27,28^, while vervet monkeys refine the correct use of alarm calls benefitting from exposure to the vocal behaviour of mature group members, who may act as positive reinforcement^29,30^. Furthermore, vervet infants exposed to intergroup vocal encounters at higher rates learn to produce the appropriate call earlier than those exposed at lower rates^31^. Although evidence of socially acquired vocalisations in adult great apes has been debated (chimpanzees, *Pan troglodytes*^32–35^), a notable exception is a recent study on wild orangutans (*Pongo pygmaeus wurmbii and P. abelii*) showing that the degree of an individual’s gregariousness predicts their vocal output and structure^36^. For comprehension or response learning, a prerequisite in both human infants^37^ and songbirds^38^ is a preference for attending to conspecific vocalisations. Across several primate species, infants’ responses to others’ vocalizations differ markedly from adults’ responses^9^. Young vervet monkeys and sooty mangabeys (*Cercocebus atys*) have the opportunity to learn the referential meaning of alarm calls by attending to the responses of adult conspecifics^30,39^. There is good evidence that adult primates can extract relevant social information from attending to calls, such as the arrival of a social partner^40^, which is arguably learned from experience of interacting with others. Critically, how immature chimpanzees acquire this capacity is not known.

Given a long period of dependency on their mother that continues after weaning, the early socialization and learning opportunities of immature chimpanzees depend almost entirely on their mothers’ social associations^41,42^, and there is rich potential for social factors to play a role in the ontogeny of vocal behaviour. For example, the appropriate use of alarm calls is acquired progressively in young chimpanzees, potentially facilitated by exposure to gaze alternations with their mothers^43^. High fission-fusion societies, such as those of chimpanzees, are characterised by a dynamic social system in which mature individuals navigate a complex network of kin and non-kin social relationships. Long-distance pant hoot calls and exchanges between parties that are out of visual contact are critical to learn to maintain spatial and social cohesion^44,45^. When compared to human infants, infant chimpanzees produce spontaneous vocalizations less frequently^46^ and very rarely produce long-distance calls^47,48^. Apart from early qualitative studies^49,50^, a limited amount of systematic research on vocal development in immature chimpanzees is available^43,51^.

In this study, we focus on the pant hoot vocalisation, a structurally complex sequence of four acoustically distinct phases typically produced in an orderly way and the most frequently uttered vocalisation by adults^47^. Pant hoots are produced spontaneously (i.e., not in response to others’ calls), as part of vocal choruses, or in response to others’ pant hoots during vocal exchanges^45,47,49,52,53^. Individuals use these long-distance contact calls to maintain social cohesion, helping to coordinate with and recruit conspecifics across a wide range of contexts, including feeding, travelling, displaying, and inter-community encounters^44,45,54^. Adult females pant hoot less frequently than adult males overall and are more likely to join others’ calls than to call spontaneously^55,56^, while immature individuals of both sexes are very rarely observed pant hooting^47^. Despite a long research tradition of this field^57^, little is known about the development of pant hoots ontogeny. Recently Soldati et al.^58^ showed that chimpanzees are capable of producing spontaneous but rudimentary pant hoot-like calls from birth, which likely undergo further developmental changes including the production of build-up and let-down phases. Bründl et al.^59^ showed that greater maternal gregariousness is positively associated with the offspring’s use of pant-hoots, but only in the first two years of life. We extend this work to examine how immature chimpanzees develop communicative competence as both signallers and receivers during pant hoot exchanges until they become sexually mature and socially independent. We initially compared the spontaneous call rate (usage learning) and response patterns (response learning) during vocal exchanges of mature and immature individuals to establish their developmental changes. Then, we examined the role of mother’s vocal and social behaviors on the immature responsiveness towards pant hoots from group members. First, we predicted that the offspring of more gregarious mothers, exposed to more social interactions, would show greater responsiveness to others’ pant hoot calls. Second, we predicted greater responsiveness with increasing age. Third, since pant hoots are most frequently used by adult males for spatial coordination and during social interactions, we predicted that immature males would show greater responsiveness than immature females. Fourth, given the importance of pant hoot chorusing between closely bonded male social partners^52^, we predicted that immature individuals would vocally respond more often when their mother vocally responded as well. Fifth, due to the effects of nearby individuals on pant hoot usage^54^, we predicted that responses would vary depending on the number of males and females in the audience.

## Results

### Comparison with mature chimpanzee spontaneous call rates

Spontaneous pant hoot production rates of mature individuals (mean = 0.48 ± 0.36 per hour) were approximately 10 times greater than those of immatures (mean = 0.05 ± 0.08 per hour) (W = 30.5, *p* < 0.001). Mature males produced pant hoots spontaneously at a higher rate (mean 0.74 = 0.35 ± per hour) than mature females did (mean = 0.23 ± 0.20 per hour). Immatures rarely produced pant hoots that were not in response to others’ pant hoots (*n* = 9 instances) and we only observed calls produced spontaneously by immature males (*N* = 4 individuals). Immature chimpanzees produced pant hoots in response (*n* = 36) four times more frequently than they produced pant hoots spontaneously (*n* = 9) (Table S1), while mature chimpanzees produced pant hoots spontaneously (*n* = 258) and in response (*n* = 278) with similar rates (Table S2).

## Responsiveness of immatures to others’ Pant hoots

We recorded a total of 554 behavioural responses to others’ pant hoots produced by 13 immature chimpanzees (Supplementary Tables S1 & S2). The most frequently recorded response was a head movement towards a pant hoot (35.7%, *n* = 198) and the second was a vocal response (9.2%, *n* = 51). All immatures responded with a head movement or a vocalisation at least once. The youngest individuals who responded with a head movement and vocalisation were observed at 16 months and 19 months of age respectively. Of all the vocal responses recorded, 70.6% were pant hoots (*n* = 36). After hearing a pant hoot from others, immatures either responded with a pant hoot alone (*n* = 24) or mothers and immatures joined each other’s pant hoot response as part of a chorus (*n* = 12), including the pant-hoot response of the youngest individual. On occasions where the mother and offspring chorused with each other, the mother initiated the call on five occasions, and the offspring on seven occasions.

## Comparison with mature chimpanzee responses

When considering the rate of head movement in response to others’ pant hoots, the difference between the full and null models was significant (LRT: χ^2^_1_ = 7.59, *p* = 0.006). Mature individuals were more likely to move their head towards a pant hoot than immatures (Table 1, Supplementary Figure S1). Individuals were also more likely to move their head when a pant hoot originated from outside the party than when it originated from within the focal individual’s party, when it was a group call than when it was a single call, in parties with a smaller number of females, and when resting than when engaged in other behavioural activities (Table 1, Supplementary Figure S1). Regarding the rate of vocal responses to others’ pant hoots, the difference between the full and null models was significant (LRT: χ^2^_1_ = 8.98, *p* = 0.003). Mature individuals were more likely to vocally respond after hearing a pant hoot than immatures (Table 2, Supplementary Figure S2). Individuals were also more likely to respond vocally when the number of females in the party was smaller, and when engaged in other behavioural activities than when resting (Table 2, Supplementary Figure S2). Pant hoot response rates of mature individuals (mean = 0.62 ± 0.32 per hour) were three times greater than those of immatures (mean = 0.21 ± 0.25 per hour). Mature males (mean = 0.63 ± 0.26 per hour) and females (mean = 0.59 ± 0.39 per hour) produced response pant hoots at similar rates.

**Table 1:** Relationship between whether or not mature or immature individuals moved their head towards a pant hoot and the investigated independent variables. Reference levels are in brackets (reference for ‘activity’ is resting).

Term	Estimate	SE	Lower CI	Upper CI	χ^2^	Z-value	*P*
Intercept	0.524	0.246	0.212	1.010			
Age (mature)	0.619	0.204	-0.080	0.633	7.585		**0.006**
Gregariousness	-0.120	0.092	-0.567	-0.076	1.618		0.203
Sex (male)	0.279	0.178	-0.305	0.061	2.365		0.124
Call from within party	-0.326	0.124	-0.610	-0.212	6.766		**0.009**
Solo call	-0.412	0.099	0.212	1.010	17.180		**< 0.001**
Number of females	-0.208	0.060	-0.324	-0.088	12.154		**< 0.001**
Number of males	0.058	0.067	-0.081	0.190	0.748		0.387
Activity: feeding	-0.317	0.120	-0.554	-0.079		-2.651	**0.008**
Activity: other	-0.511	0.157	-0.829	-0.210		-3.249	**0.001**
Activity: social	-1.257	0.165	-1.572	-0.921		-7.622	**< 0.001**

CI: confidence interval. Control variables are in italic. Significant results are depicted in bold.

**Table 2:** Relationship between whether or not mature or immature individuals produced a vocal response to a pant hoot and the investigated independent variables. Reference levels are in brackets (reference for ‘activity’ is resting).

Term	Estimate	SE	Lower CI	Upper CI	χ^2^	Z-value	*P*
Intercept	-2.821	0.319	-3.408	-2.127			
Age (mature)	0.798	0.270	0.196	1.314	8.978		**0.003**
Gregariousness	-0.082	0.116	-0.308	0.148	0.504		0.478
Sex (male)	0.269	0.225	-0.188	0.716	1.410		0.235
Call from within party	-0.242	0.163	-0.546	0.093	2.258		0.133
Solo call	-0.167	0.122	-0.405	0.073	1.876		0.171
Number of females	-0.266	0.084	-0.423	-0.091	10.756		**0.001**
Number of males	0.076	0.084	-0.086	0.247	0.814		0.367
Activity: feeding	0.144	0.195	-0.230	0.547		0.736	0.462
Activity: other	0.291	0.148	-0.004	0.576		1.966	**0.049**
Activity: social	-0.219	0.251	-0.701	0.325		-0.875	0.381

CI: confidence interval. Control variables are in italic. Significant results are depicted in bold.

## Factors affecting head movement in immatures

**Fig. 1:**
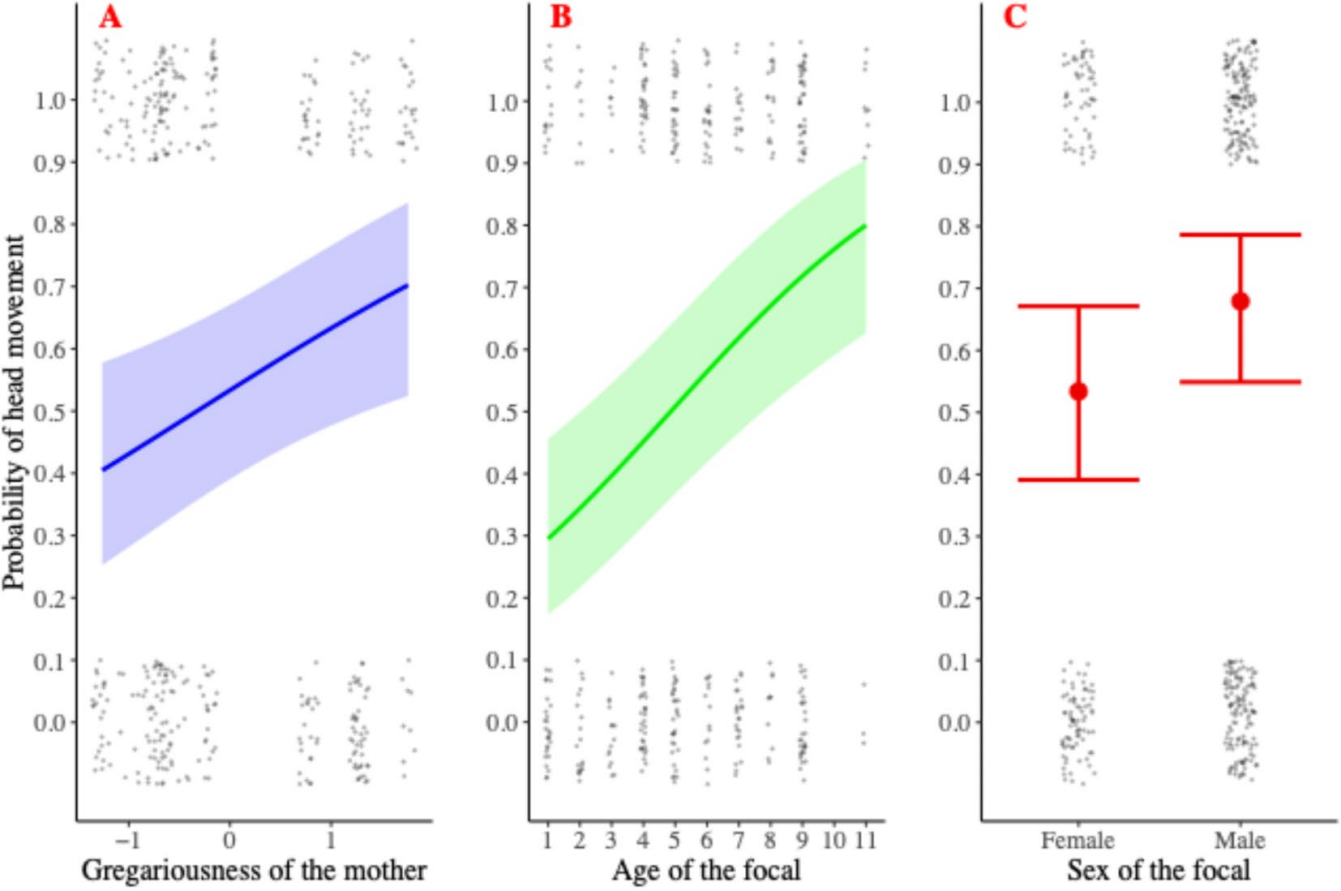
Likelihood of moving the head towards the source of the pant hoot depending on (**A**) maternal gregariousness (numerical), (**B**) offspring’s age (numerical), and (**C**) offspring’s sex (categorical). Gregariousness values were z-standardized. Confidence bands and bars illustrate the standard errors (95%). Note that raw data, represented here with dots spread around the dependent variable values of either 0 or 1, do not express the influence of other factors included in the model.

The difference between the full and null models was significant (LRT: χ^2^_4_ = 11.14, *p* = 0.025). We found that the immature offspring of more gregarious mothers were more likely to move their head towards the caller than the immature offspring of less gregarious mothers (Table 3; Fig. 1a). There was a positive relationship between the age of immatures and the likelihood of the focal moving their head towards a pant hoot (Table 3; Fig. 1b). Males were more likely to do this than females (Table 3; Fig. 1c). Immatures were also more likely to move their head during social behavioural activities (LRT: χ^2^_4_ = 20.93, *p* < 0.001) and after hearing a group call (Table 3, Supplementary Figure S3).

**Table 3:** Relationship between whether or not the focal individual moved their head towards the pant hoot produced by another individual and the investigated independent variables. Reference levels are in brackets (reference for ‘activity’ is resting).

Term	Estimate	SE	Lower CI	Upper CI	χ^2^	z-value	*P*
Intercept	-1.097	0.386	-1.816	-0.233			
Gregariousness	0.411	0.150	0.075	0.690	5.758		**0.016**
Age	0.226	0.053	0.104	0.322	9.486		**0.002**
Sex (male)	0.613	0.251	0.080	1.117	5.373		**0.020**
Mother chorus	0.299	0.601	-1.134	1.499	0.250		0.617
Call from within party	0.284	0.321	-0.382	0.948	0.784		0.376
Solo call	-0.479	0.222	-0.917	-0.003	4.713		**0.030**
Number of females	0.047	0.121	-0.205	0.292	0.152		0.697
Number of males	0.078	0.131	-0.209	0.343	0.361		0.548
Activity: feeding	-0.101	0.286	-0.677	0.489		-0.354	0.723
Activity: other	-0.785	0.420	-1.586	0.154		-1.868	0.062
Activity: social	-1.205	0.333	-1.826	-0.461		-3.623	**< 0.001**

CI: confidence interval. Control variables are in italic. Significant results are depicted in bold.

## Factors affecting vocal response in immatures

**Fig. 2:**
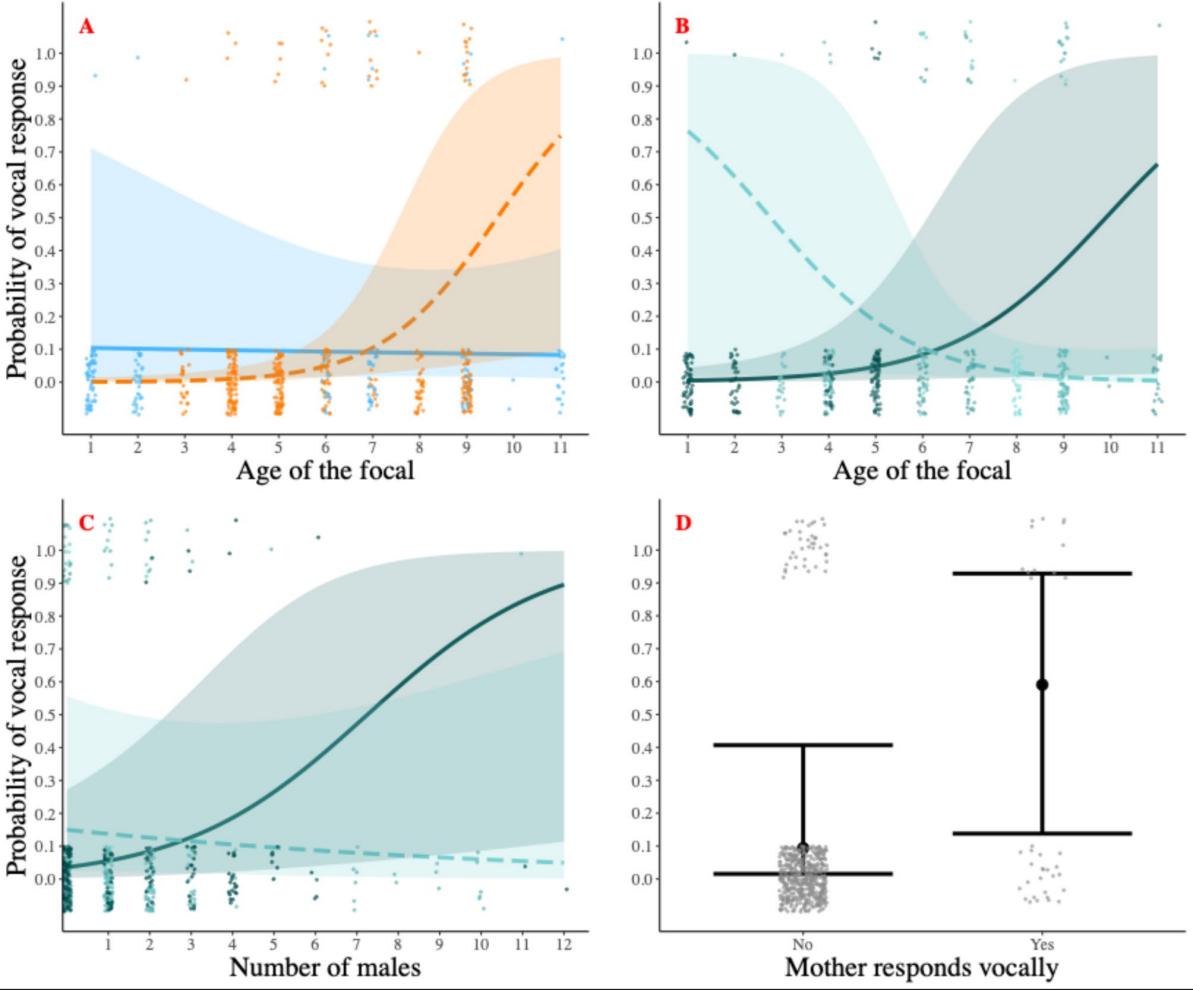
Likelihood of vocally responding to a pant hoot depending on (**A**) the interaction between age (numerical) and sex (categorical) of the offspring (orange: male; blue: female), (**B**) the interaction between the age of the offspring (numerical) and maternal gregariousness (numerical; solid sea green: +1 SD above the mean; dashed sea green: -1 SD below the mean), (**C**) the interaction between the number of males in the party (numerical) and maternal gregariousness (numerical; solid sea green: +1 SD above the mean; dashed sea green: -1 SD below the mean), and (**D**) whether the mother joined the vocal response (categorical). Confidence bands and bars illustrate the standard errors (95%). Note that raw data, represented here with dots spread around the dependent variable values of either 0 or 1, do not express the influence of other factors included in the model.

The difference between the full and null models was significant (LRT: χ^2^_7_ = 31.52, *p* < 0.001). Immatures were more likely to vocally respond to pant hoots when their mothers participated in a chorus (Table 4; Fig. 2d). The confidence intervals indicate that the following significant results should be interpreted with more caution, even if additional tests showed that no individual was responsible for the model output, the model was not overfitted, and it had explanatory power (See Supplementary Information). We found an interaction effect between the age and sex of immatures, meaning that male individuals were more likely to vocally respond when older (Table 4; Fig. 2a). We found an interaction effect between the age of immatures and maternal gregariousness, meaning that the offspring of more gregarious mothers were more likely to vocally respond when older, and the offspring of less gregarious mothers were more likely to vocally respond when younger (Table 4; Fig. 2b). We also found an interaction effect between the maternal gregariousness and the number of males in the party: the offspring of more gregarious mothers were more likely to respond vocally as the number of males increased while the offspring of less gregarious mothers were more likely to vocally respond as the number of males decreased (Table 4; Fig. 2c).

**Table 4:** Relationship between whether or not the focal individual produced a vocal response to the pant hoot produced by another individual and the investigated independent variables. Reference levels are in brackets (reference for ‘activity’ is resting).

Term	Estimate	SE	Lower CI	Upper CI	χ^2^	z-value	*P*
Intercept	-2.269	0.967	-4.032	0.416			
Gregariousness	-0.386	0.410	-1.195	0.714			
Age	-0.061	0.517	-1.453	1.160			
Sex (male)	-1.024	0.860	-3.128	0.840			
Mother chorus	2.630	0.659	0.787	3.792	17.123		**< 0.001**
Call from within party	0.436	0.548	-0.694	1.723	0.621		0.430
Solo call	-0.218	0.375	-0.976	0.622	0.341		0.562
Number of females	-0.480	0.256	-0.933	0.181	3.696		0.055
Number of males	0.353	0.243	-0.208	1.048			
Activity: feeding	0.634	0.481	-0.642	1.553		1.318	0.186
Activity: other	0.614	0.592	-0.797	1.899		1.037	0.298
Activity: social	-0.200	0.624	-1.619	1.306		-0.320	0.751
Age*Sex (male)	2.286	1.050	-0.322	4.458	4.411		**0.035**
Age*Gregariousness	1.759	0.791	-0.435	3.237	4.655		**0.030**
Gregariousness* Number of males	0.501	0.235	-0.228	1.066	4.554		**0.024**

CI: confidence interval. Interactions are represented by an asterisk between variables. Control variables are in italic. Significant results are depicted in bold.

### Maternal gregariousness and vocal exposure

**Fig. 3:**
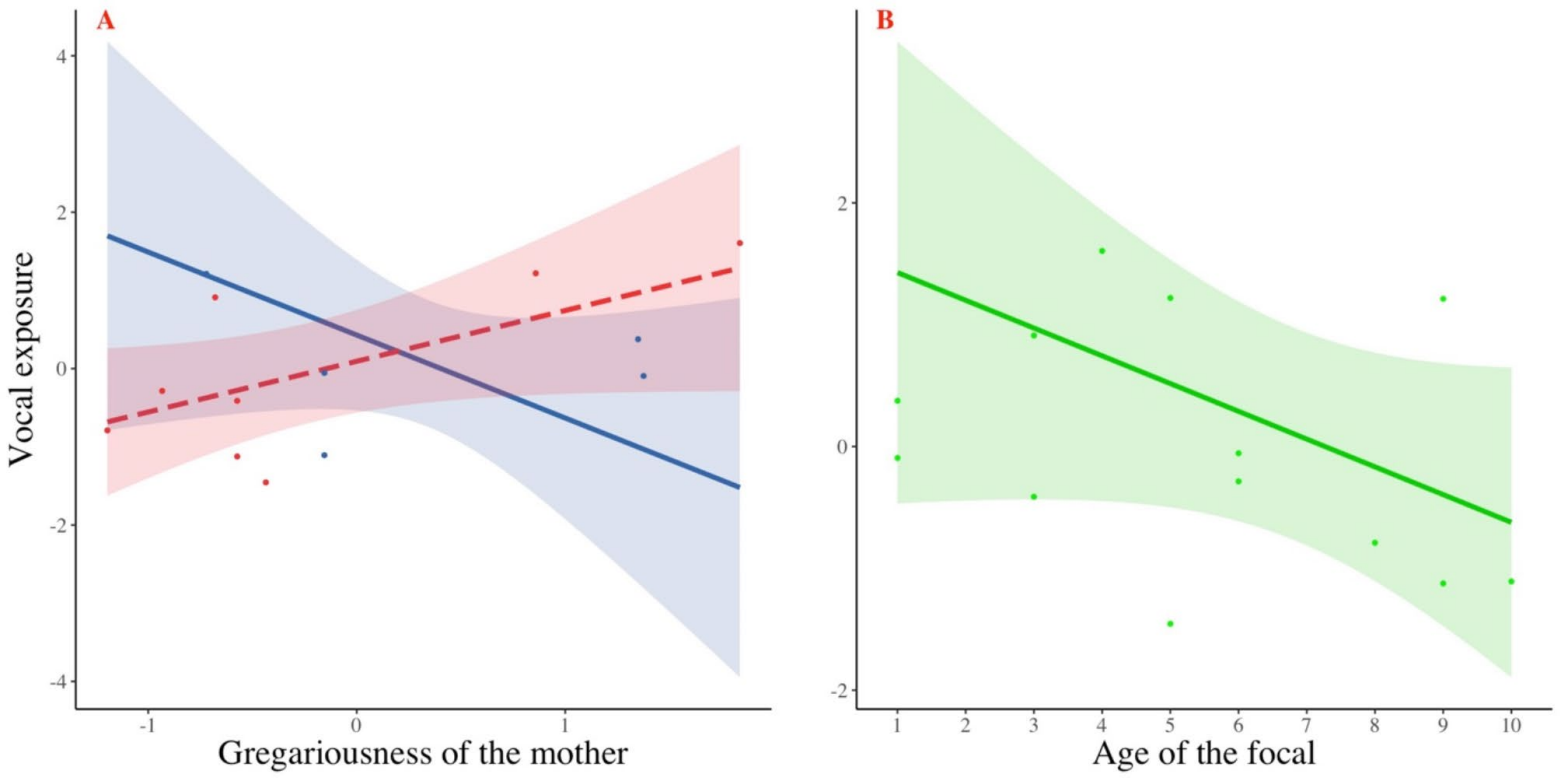
Vocal exposure to pant hoots of immature individuals depending on (**A**) the gregariousness value of their mother (numerical; red: males; blue: females), and (**B**) the age of the focal (numerical). Gregariousness and vocal exposure values were z-standardized. Confidence bands illustrate the standard errors (95%). Note that raw data, represented here with dots spread around the dependent variable values of either 0 or 1, do not express the influence of other factors included in the model.

The difference between the full and null models was significant (LRT: χ^2^_2_ = 10.06, *p* = 0.007, R^2^_m_ = 0.59, R^2^_c_ = 0.38). We found an interaction effect between the sex of the offspring and maternal gregariousness, meaning that the male offspring of more gregarious mothers were more exposed to pant hoots than other immatures (Table 5; Fig. 3a). We also found that younger individuals were more exposed to pant hoots than older individuals (Table 5; Fig. 3b), although the confidence intervals indicate that this effect may not be reliable.

**Table 5:** Relationship between the offspring’s exposure to pant hoots and the investigated independent variables. Reference levels are in brackets (reference for ‘activity’ is resting).

Term	Estimate	SE	Lower CI	Upper CI	T-value	*P*
Intercept	0.429	0.417	-0.534	1.391		
Gregariousness	-1.062	0.663	-2.590	0.467		
Age	-0.681	0.376	-1.547	0.186	-1.811	**0.034**
Sex (male)	-0.335	0.513	-1.519	0.848		
Gregariousness*Sex (male)	1.711	0.659	0.191	3.229	2.597	**0.005**

CI: confidence interval. Interaction is represented by an asterisk between variables. Control variables are in italic. Significant results are depicted in bold.

## Discussion

We found that immature chimpanzees rarely produce pant hoots spontaneously, and considerably less often than mature individuals. Instead, they usually respond to the pant hoots of others using different call types, the majority being pant hoots, as part of vocal exchanges. Immatures also respond by moving their heads in the direction of the caller, although less often than mature individuals. The youngest individual we observed producing a pant hoot spontaneously was three years old, while the youngest individual who produced a pant hoot response was 19 months old. We found that more gregarious, older, and male immatures are more likely to move their head towards the calls of others compared to less gregarious, younger, and female immatures. Furthermore, more gregarious and older immatures, older male immatures, and more gregarious immatures in the presence of larger male audiences are more likely to respond vocally compared to younger and female immatures, and in the presence of smaller male audiences. Immatures are more likely to respond vocally when their mother also respond vocally to the same call. Finally, exposure to others’ vocalisations is higher in the male offspring of more gregarious mothers.

Overall our observations suggest that the vocal development of chimpanzees is progressive, consistent with the idea that the acquisition of social calls is less hard-wired and likely slower than that of alarm calls^19^ and that the period of acquisition of communicative skills is extended in great apes^60^. We observed that spontaneous pant hoot use was very rare in both infants and juveniles, only becoming frequent in mature individuals where spontaneous and response pant hoots were produced at comparable rates. One possibility is that the increase in spontaneous calling rate coincides with individuals engaging independently from their mother in social contexts mediated by pant hoots, where spontaneous pant hoots could be used to elicit responses from others to coordinate joint movement or as a form of social bonding. We also found that there was a positive effect of the age of individuals on head movements and vocal responses (males and gregarious individuals only). Immature chimpanzees were capable of producing both behavioural responses at the youngest age recorded but reached adult levels of responsiveness as late juveniles. Chimpanzees produce rudimentary pant hoots from birth but only develop adult-like phase structures upon reaching full maturity^58^. Together with our observation of an increase in spontaneous and response pant hoots when transitioning to maturity, one possibility is that chimpanzees require longer practice or exposure to others’ calls to develop complex vocal sequences, similarly to the development of complex song sequences in songbirds^61^ and of complex social calls in mice^62^ and lemurs^63^. While physical maturation contributes to the ontogeny of vocal behaviours^64^, our observations indicate that the vocal ontogeny of great apes is less constrained and genetically fixed than previously assumed. Nevertheless, longitudinal, as opposed to cross-sectional, studies using observations from birth until adult-hood are necessary to fully determine the developmental trajectory of these vocal behaviours. While our findings included a relatively broad sample of inviduals, the level of inter-individual variation observed suggests that longitudinal designs may be particularly well suited to investigating. Furthermore, the ontogeny of socially used, long and short distance calls such as pant hoots may also depend on the development of cognitive constructs, such as social awareness^44,54^. We observed that the responses of immatures varied depending on the audience composition, the type of call, and the context, providing further evidence that primate responses are highly flexible^65^. As non-focal callers were typically out of sight, we were not able to explore the effects of the caller’s identity or their affiliative relationship on the responses of immatures. Future studies are necessary to investigate how additional fine social factors may impact the ontogeny of responses.

Our study supports the idea that immature chimpanzees develop vocal behaviours by socially interacting with their conspecifics. By spending more time in groups, gregarious mothers increase the exposure of their offspring to social and vocal interactions. Mature individuals and particularly males produce pant hoots most often^55,56^ and can act as ‘models’ for younger individuals. The fact that the offspring of more gregarious mothers responded vocally more often in the presence of males further corroborates the idea that younger individuals have more opportunities to reply by calling when spending time with males. Greater social opportunities to learn associations between vocalisations and appropriate usage and responses may help to increase the speed of vocal development. The need for experience of others’ vocalisations is not widespread among animals and an important role of auditory feedback has been demonstrated in the development of a few non-human animals regarded as vocal learners, including songbirds, dolphins and whales, bats, and elephants^8,12,17,61^. Our findings support the idea that the vocal ontogeny of chimpanzees should not be regarded as qualitatively different from that of songbirds and human for instance, despite chimpanzees possessing considerably less sophisticated vocal production learning abilities^17,38^. Furthermore, with growing evidence of diverse range of vocal learning behaviours across several animal species, we also believe that the dichotomous view of “haves and have-nots” should be abandoned in favour of a more nuanced and continuous approach to the study of vocal learning capacities^12^.

In addition to providing more exposure and opportunities to learn from others through association patterns, mothers positively affected the vocal responses of their offspring by joining in chorused calls. Offspring and mother pairs might call together to signal their bond strength, similarly to what has been observed in adult males^52^. Chorusing might also facilitate the learning process, similarly to how parents’ vocal feedback improves appropriate usage learning in marmosets^18^ and in gibbons^66^. Because we were not able to investigate the temporal order of callers during chorused calls due to the low number of chorused responses, further studies are needed to clarify the role of chorusing during the vocal development of chimpanzees. Nevertheless, the fact that we did not observe individuals directing vocalisations at each other is potentially at odds with evidence from human caregivers, who typically use vocalisations accompanied by head orientation and directed toward young receivers to facilitate language acquisition^16^. Similar evidence in great apes is so far lacking^6^, but might be more easily detected in a short-distance interactions^54^ as opposed to long-distance exchanges. Determining whether chorusing represents an intermediate developmental stage between directed and broadcasted vocalisations requires further investigation.

Our observations are in line with the idea that mothers and other mature individuals in the group do not actively or directly ‘teach’ immature chimpanzees their vocal behaviour (*sensu*^67^). Instead, our findings are more consistent with the ‘master-apprenticeship’ hypothesis, which posits that young chimpanzees acquire behaviours through repeated exposure and observation of a tolerant model in close proximity^68^. In Western chimpanzees of the Taï Forest, the offspring of mothers who had been highly gregarious use pant hoots more frequently, but this was only observed during the first 2-years of life^59^. Because we found that greater maternal gregariousness increases the probability of vocal responses in juvenile individuals up to 11-years old, it is possible that these differences are related to greater variation in Eastern female chimpanzees’ association patterns and lower social cohesion than their Western counterparts^69^. Because pant hoots are used to mediate fission-fusion events and Eastern communities are larger in size than Western ones^69^, it is likely that young Eastern chimpanzees experience greater variation in social and vocal exposure. Thus, data collected from groups that are particularly small and cohesive may not generalise across the species as a whole. Our findings show the importance of incorporating a wide range of the diverse social structures and patterns of association that characterise chimpanzee fission-fusion social system, particularly when they are used in a comparative framework. Furthermore, we show that vocal ontogeny is an ongoing process that persists well into the juvenile phase and that attending to and interacting with mothers and male group members during vocal exchanges are also key drivers. Early social interactions with conspecifics also play an important role in the acquisition of gestural communication in chimpanzees^70^, which, similar to their vocal repertoire, some authors argue is based on a largely innate, species-typical repertoire of signals that is then used flexibly^71^. Overall, greater social inputs provide young chimpanzees with a head start for their communicative skills.

The predominant role of pant hoots in the social lives of adult male chimpanzees is reflected in the greater responsiveness and spontaneous usage we observed in both mature and immature males, particularly in older immature individuals. The importance of social calls in the relationships and interactions between male chimpanzees likely explains the sex differences during its ontogeny. As compared to immature females, males experience more social exposure and opportunities to learn from others due their mothers typically associating and interacting more with group members and particularly with adult males^41^. Similarly, immature males may develop their pant hoots earlier due to greater exposure to the vocal behaviour of group members. Our findings are consistent with the idea that the development of social skills, including vocal behaviour^59^, occurs ontogenetically earlier in male chimpanzees^72^, and that these differences between sexes are in-part explained by the different adaptive sex-dependent pressures in adulthood. It is important to note that sex differences might be differently expressed in populations where females are more gregarious^69^, restricted to specific developmental phases, or have a combined effect with other factors, as seen during human speech development^73^. Investigating the effects of other social factors (e.g. centrality in a social network or dominance) in future studies could provide further insights into how sociality shapes chimpanzee vocal ontogeny.

In conclusion, we found that the ontogeny of chimpanzee vocal usage and comprehension are mediated by social and individual factors, in line with the hypothesis that some form of social learning enhances the development of vocal communicative skills, even in species with apparently limited vocal production learning^17^. Extending this research across chimpanzee communities and great ape species would further clarify the ways in which variation in social dynamics and group structures can drive differences in hominid vocal development. In addition, given new findings that suggest greater flexibility in great ape vocal production than previously assumed^20^, there is substantial promise for studies exploring how vocal ontogeny is socially-mediated and the potential development of dialects^34^. Finally, whether the development of the structure and order of pant hoot components is also socially mediated remains an interesting avenue for future research, particularly given the parallels between the combinatorial structures used by primates and those present in human language^21^. In sum, our findings do not support theories of primate vocal behaviours as fixed and impervious to social influence and are instead consistent with the emerging view that the origin of human language was a continuous evolutionary process built upon precursors, an increasing number of which can be found in the vocal communication of modern ape species.

## Methods

### Ethics & inclusion statement

One of the co-authors of this study is a local researcher and field assistant with extensive expertise in the study site and species. The project adhered to the ASAB guidelines for the treatment of animals during behavioural studies. It was approved by the Uganda Wildlife Authority (UWA/COD/96/5), the Uganda National Council for Science and Technology (NS 637) and the research ethics committees of the Universities of St Andrews and Neuchâtel (38/2019-B; No 171). Data collection was terminated on 17 March 2020, due to the Covid-19 pandemic (UWA ref: EDO/73/01), to avoid putting the health of the animals at risk.

### Study site

The study was conducted with Eastern chimpanzees of the Sonso community (*Pan troglodytes schweinfurthii*) of the Budongo Forest in western Uganda. At the beginning of the study (September 2018) the community was composed of 74 individuals (11 adult males, 25 adult females, 15 sub-adults, 8 juveniles, and 15 infants). At the end of the study (March 2020) the community was composed of 68 individuals (9 adult males, 26 adult females, 15 sub-adults, 9 juveniles, and 8 infants) (Supplementary Table S3).

### Study subjects

We used age categories following Reynolds^74^ of infants (0–4 years), juveniles (5–9 years), sub-adults (males: 10–15 years, females: 10–14 years), and adults (males: >16 years, females: >15 years). Study subjects were selected to obtain balanced samples of each infant and juvenile age category in the community: 2 of 4 available female infants, 4 of 7 male infants, 2 of 4 female juveniles, 4 of 4 male juveniles, all of which were dependent from their mother (i.e., mother and offspring were always in the same association party) and are collectively referred to as ‘immatures’ hereafter (Supplementary Table S1). In addition, one female aged 10 at the beginning of the study was considered immature as during data collection she was dependent on her mother (always travelled with and engaged in activities in the presence of her mother). Instead, we considered individuals who already spent time with other group members in parties independently of the presence of their mother as mature and refer to them collectively as ‘matures’ hereafter^75^. We collected data on 25 mature individuals (13 males, 12 females) (Supplementary Table S2). Because collecting data longitudinally was not possible, we opted for a cross-sectional study design. Data were collected between September 2018 and March 2020 and we assigned the age of the focal (in years) to each data point, where for the same individual we may have data across the observation period (Table S1).

### Definition of Pant hoot vocalisations

**Fig. 4:**
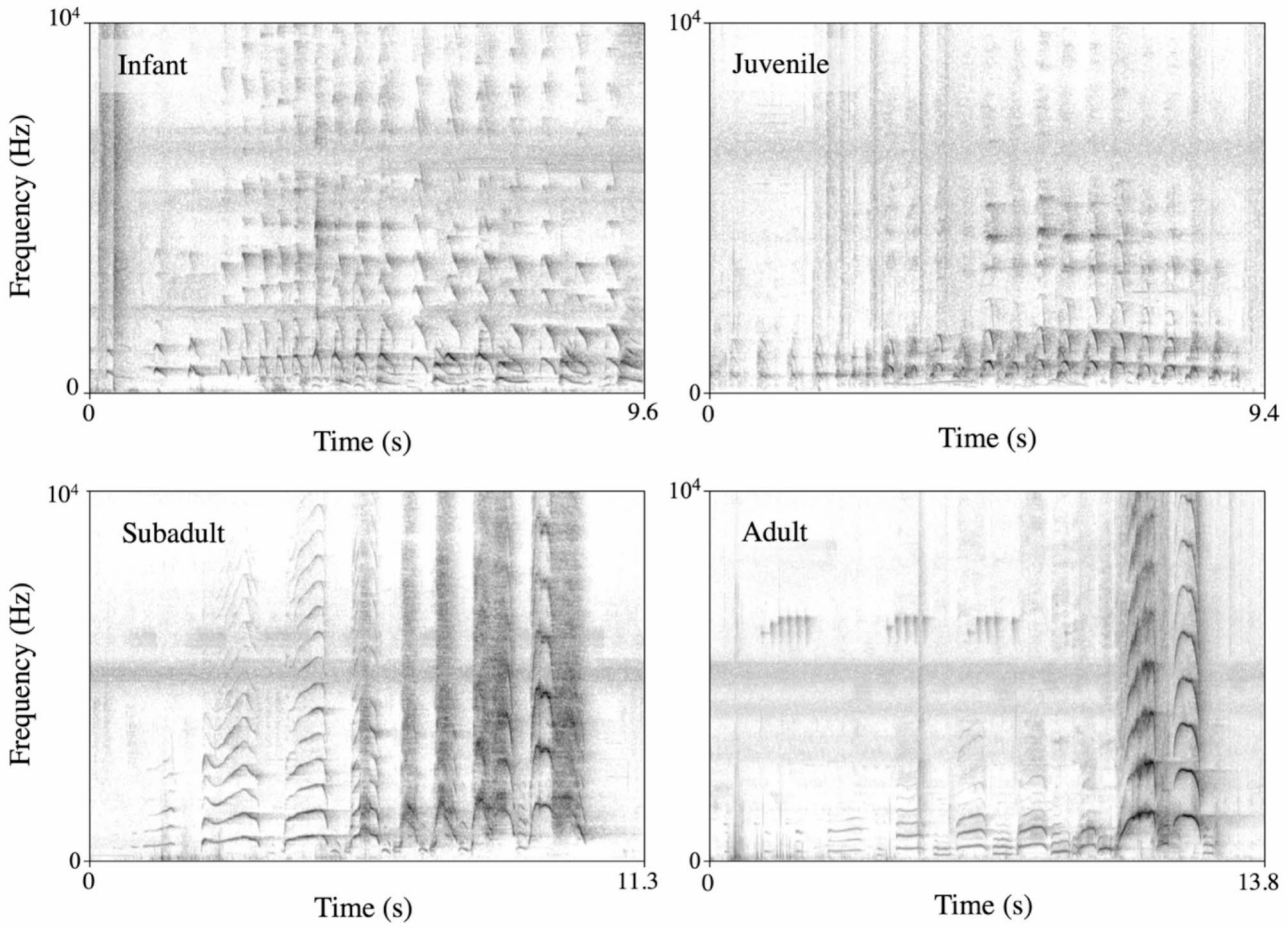
Spectrographic representations of pant hoots produced by an infant, a juvenile, a subadult, and an adult chimpanzee from the Sonso community.

Pant hoots are long-distance vocal sequences composed of up to four acoustically distinct phases, typically produced during traveling and feeding events^47,57^ (Fig. 4). Each phase contains one or more acoustically similar voiced elements produced in the following order: Introduction, Build-up, Climax, and Let-down. Because pant hoot phases can be omitted or produced in isolation^54^, we considered a pant hoot when we heard a caller produce at least one of the four phases. Another reason for adopting this criterion is that, although the climax is never produced in isolation^54,55^, for most distant pant hoots we were only able to hear the climax phase, the highest amplitude phase.

### Data collection

Data were collected between September and December 2018, February and July 2019, and November 2019 and March 2020, for a total of 15 months. We used focal animal sampling as the main method of data collection, following a different study subject each day from 0700 to 1630 h, approximately 5.5 days a week. We avoided following the same individual on consecutive days while balancing sampling time across individuals. Each focal animal was followed for at least three separate days. We calculated the duration of focal follows by taking into consideration periods of time when the face of the focal animal was visible to ensure reliable observation of behavioural changes. Consequently, focal durations reported here may be more conservative. We collected a total of 170.7 h of observations with a mean of 13.1 h per immature individual (SD = 4.4, range 6.5–21.2; Supplementary Table S1) and a total of 451.9 h of observations with a mean of 18.1 h per mature individual (SD = 8.7, range 8.7–34.6; Supplementary Table S2). We recorded all the observational data on a portable device (Samsung Xplorer 4) using a custom-built CyberTracker database (version 3.496), which automatically paired it with the time of occurrence. We recorded whenever we heard a pant hoot and, if no other calls were produced by other individuals during at least 15 s after the pant hoot we heard, when the focal individual produced a pant hoot we later coded it as a ‘spontaneous’ call. When another individual produced a pant hoot, we recorded the behavioural changes of the focal after the call (see *Behavioural responses* below). When the pant hoot heard by the focal was a call that was subsequently joined by one or more individuals to form a chorus (hereafter ‘group’ call), we noted the behavioural responses that followed the first call heard.

### Behavioural responses

We recorded ‘head movement’ responses when the focal individual moved the head in the direction of a pant hoot for ≥ 1 s within five seconds after the termination of the pant hoot heard, and the movement was at least a 45 degree change from the head position before the call. We excluded instances where the head of the focal individual was already oriented within a 45 degrees angle from the call source. We recorded ‘vocal responses’ when within five seconds after hearing a pant hoot the focal individual produced a vocalisation^53^. We recorded the type of vocalisation produced following the repertoire by Slocombe and Zuberbühler^76^. Because in some instances it was possible to assess whether the focal produced a vocal response but not the head orientation with certainty, the number of vocal responses is larger than the number of head movements (see Statistical analyses). As in a previous study on chimpanzee responses to calls^53^, we used a five-second threshold to maximize the inclusion of responses that may require processing time by receivers engaged in different behavioural activities. To minimize the chances of recording responses to other signals, we did not record behavioural responses if we heard other stimuli between the pant hoot and the response.

We noted whether the call heard by the focal individual was produced by a single individual or multiple individuals (solo vs. chorus), whether the mother of the focal produced a vocal response (if the mother was not visible we excluded the data), and whether the call was uttered from within the party of the focal individual. Party was defined as all mature individuals present in the visual range, which corresponds to a radius of approximately 35 m^77^. We excluded calls estimated to be produced by neighbouring chimpanzee communities (on the basis of distance, location, and the chimpanzees’ reaction). We continuously scored the behavioural activity of the focal using four categories: (1) resting, which included self-directed grooming; (2) feeding; (3) social, which included grooming and social play; (4) other activity, which included traveling, aggression, and solitary play. We provide raw data of the recorded behaviours in the Supplementary Information (Table S1, S2).

### Gregariousness

We defined “gregariousness” as the probability of finding an individual in a party with other chimpanzees^78^. Four BCFS field assistants collected data on party composition every 15-minute intervals during the period December 2017 – June 2020 while conducting focal follows on a different mature individual each day as part of long-term data collection. To establish gregariousness, we calculated the number of 15-minute scans in which a mature individual was recorded in a party with other mature individuals. We then calculated the total number of occurrences across all mature study subjects. In Eastern chimpanzees, fission-fusion events occur more frequently than in Western chimpanzees and less gregarious (i.e., peripheral) females are most often recorded by observers when present in large parties^69,78^. Therefore, we excluded focal data from our calculations and only included instances where an individual was observed with others while not being the focal animal to avoid overestimating the gregariousness values of less gregarious mature females as well as to control for unbalanced sampling efforts across individuals^78^. Then, we divided the total number of occurrences by the number of times they were observed while not being the focal animal and multiplied by 100 to obtain the probability of finding an individual in a party with other chimpanzees. Values varied between 0 (always alone) and 100 (always with others), with lower values indicating individuals who are less likely to spend time in a group, while higher values indicate individuals who are more likely to spend time in a group. Because all immature chimpanzees considered in this study did not associate with other group members independently of their mother, their gregariousness value was the same as that of their mother. The maternal gregariousness of immature individuals had a mean value of 11.5 (SD = 4.9, range 5.6–20.7; Supplementary Table S1) and the gregariousness of mature individuals had a mean value of 17.7 (SD = 6.2, range 5.8– 29.2; Supplementary Table S2). Because females are typically more peripheral than males, and peripheral females tend to isolate more^69,78^, we included data from a longer period of time that is largely overlapping with the period during which we conducted observations, which allowed us to calculate gregariousness values reliably for all individuals. During this period, the dominance hierarchy of the Sonso community remained relatively stable^54^.

### General procedure for linear models

We z-transformed the distribution of the quantitative variables into a distribution with a mean of 0 and standard deviation (SD) of 1 to improve the accuracy of parameter estimates. Before fitting the models, we removed observations that contained missing values (NAs) in one or more of the predictor or response variables. To assess the significance of the test predictors, we compared each model with a ‘null’ model comprising only the intercept, control variables, and random effects using a likelihood-ratio test (LRT)^79^. We assessed the variance inflation factor of the variables (VIF) to measure collinearity and accepted it when < 4.0 ^80^ using the ‘performance’ package^81^ (version 0.5.1). We calculated p-values using LRT comparing each model with the respective null model using the ‘drop1’ function of the package ‘stats’^82^ (version 4.0.2). In the linear models, we checked whether the residuals were normally distributed and homogenous by inspecting a scatterplot and quantile-quantile plot of the residuals as a function of the fitted values. In the generalised linear mixed models (GLMM) we included the identity of the focal as a random effect to control for replicated observations and an unbalanced dataset. To detect the presence of influential observations (i.e., outliers), we measured the Cook’s distance using the package ‘performance’^81^ (version 0.10.2.4). All analyses were carried out using R^82^ (version 4.1.2). Figures were created using the R package ‘jtools’^83^ (version 2.2.1). The complete model structures and figures of additional variables can be found in the Supplementary Information. The dataset and code used in the analyses are available online (https://osf.io/ze82d/?view_only=c58e1e88758a4a2bb7801c89991bc92b).

### Comparison with mature chimpanzee spontaneous call rate

To examine differences in the production of spontaneous pant hoots, we compared the call rates of mature and immature individuals (Supplementary Tables S1 & S2). We calculated call rates for each individual dividing the number of spontaneously produced pant hoots by the number of focal hours. Given the small sample size, we performed a Shapiro-Wilk test to check whether the distribution of the data departed significantly from normality. The data from mature chimpanzees did not significantly depart from normal distribution (W = 0.935, p-value = 0.115). Because the data from immatures did so (W = 0.653, p-value < 0.001), we used a non-parametric Wilcoxon rank-sum test.

### Comparison with mature chimpanzee responses

To examine differences in the behavioural responses of mature and immature chimpanzees, we created two GLMMs with a binomial error structure using the R package ‘lme4’^84^ (version 3.6.3). In the first model, as the dependent variable we included whether or not (0/1) the focal individual moved their head towards the pant hoot they heard (*n* = 1995 pant hoots, 1269 of which had a head movement). In the second model, as the dependent variable we included whether or not (0/1) the focal individual vocally responded to the pant hoot they heard (*n* = 2502 pant hoots, 325 of which had a vocal response). In both models, we included whether the individuals were mature or immature (categorical with two levels; reference level: mature), the gregariousness (numerical), and sex (categorical with two levels; reference level: male) of the focal as independent variables. Because pant hoots are flexibly used depending on the social context and affected by audience composition^54^, we included as control variables whether the pant hoot was produced within or outside the focal’s party (categorical with two levels), whether the call was a solo or group call (categorical with two levels), the behavioural activity of the focal (categorical with four levels; reference level: resting), and the number of females and males in the party (numerical). There was no collinearity between the examined independent variables (maximum VIF: 1.55 and 1.59) and the dependent variables were not over-dispersed (dispersion ratio: 0.97 and 0.92).

### Behavioural responses of immature individuals

To investigate which factors impact the behavioural responses of immatures to others’ pant hoots, we created two GLMMs with a binomial error structure. In the ‘head movement model’ we considered as the dependent variable whether or not (0/1) the focal individual moved their head towards the pant hoot (*n* = 401 pant hoots, 198 of which had a head movement). Second, we created a ‘vocal response’ model where the dependent variable was whether or not (0/1) the focal individual produced a vocalisation in response (*n* = 549 pant hoots, 51 of which had a vocal response). In both models, we included as independent variables the maternal gregariousness (numerical), age of the focal (numerical in years), sex of the focal (categorical), and whether the mother joined or not the vocal response (categorical; ‘mother chorus’ hereafter). We included the same control variables as in the previous models. We initially included a three-way interaction between maternal gregariousness, age, and sex, to control for confounding factors given that 1) mothers with male offspring are more gregarious^41^, 2) the association of offspring with adult group members changes during ontogeny^42^, and 3) behavioural differences between the sexes can appear at different ontogenetic stages^75^. We also included an interaction between both the number of males and females with the maternal gregariousness because more gregarious mothers tend to spend more time in larger parties. We then removed non-significant interactions (estimates with *P* > 0.05) one at a time. We did not interpret the main effects that were part of an interaction when the interaction was statistically significant because the effect on the response of one variable was conditional on the state/value of the other and would thus be unreliable^85^. There was no collinearity between the examined independent variables (maximum VIF: 1.95 and 1.71) and the dependent variables were not over-dispersed (dispersion ratio: 1.04 and 0.66).

### Maternal gregariousness and vocal exposure

We tested whether maternal gregariousness was related to the offspring’s rate of exposure to others’ pant hoots. We created a linear model in which we used the number of pant hoots heard by the immatures per hour of focal following (‘vocal exposure’ hereafter; mean 3.36 ± 0.91) as the dependent variable and the same independent variables as in the previous models. There was no collinearity between the examined independent variables (maximum VIF: 1.72).

## Electronic supplementary material

Below is the link to the electronic supplementary material.

Supplementary Material 1

## Data Availability

Additional information has been uploaded as part of the Supplementary Information. The data and code used in the analyses are available using the following link: https://osf.io/ze82d/?view_only=c58e1e88758a4a2bb7801c89991bc92b.
