# Supplementary material for: Social and individual factors mediate chimpanzee vocal ontogeny
Source: Sci Rep. 2025 Mar 12;15:8529. doi: 10.1038/s41598-025-93207-x (PMC11903896; doi:10.1038/s41598-025-93207-x)
Supplement: Supplementary file 1 — Supplementary Material 1 [file 41598_2025_93207_MOESM1_ESM.docx]

**Supplementary Information**

**Social and individual factors mediate chimpanzee vocal ontogeny**

Supplementary Methods

*Study subjects*

Table S1 List of immature individuals considered in our study ordered by gregariousness

| *ID* | *Mother ID* | *Age (years)* | *Sex* | *Head movement* | *Vocal responses* | *Spontaneous pant hoots* | *Focal duration (h)* | *Gregariousness* |
| --- | --- | --- | --- | --- | --- | --- | --- | --- |
| JB | JL | 8, 9 | M | 20 (N = 45) | 2 (N = 56), 2 PH | 0 | 21.2 | 5.55 |
| GF | GL | 6, 7 | M | 13 (N = 22) | 13 (N = 33), 9 PH | 1 | 10 | 6.87 |
| TW | TJ | 9 | F | 6 (N = 16) | 4 (N = 30), 3 PH | 0 | 6.5 | 7.92 |
| KQ | KA | 3, 4 | M | 17 (N = 38) | 1 (N = 48), 1 PH | 1 | 11.7 | 8.14 |
| MZ | ML | 3, 4 | M | 6 (N = 28) | 3 (N = 36), 3 PH | 0 | 13.4 | 8.66 |
| MB | ML | 9 | M | 30 (N = 36) | 12 (N = 50), 4 PH | 3 | 20.1 | 8.66 |
| KJ | KW | 5, 6 | M | 11 (N = 22) | 2 (N = 31), 1 PH | 4 | 16.2 | 9.35 |
| HM | HT | 6, 7 | F | 16 (N = 34) | 6 (N = 46), 5 PH | 0 | 13.9 | 10.75 |
| HR | HT | 10, 11 | F | 9 (N = 12) | 1 (N = 21), 1 PH | 0 | 8.5 | 10.75 |
| OZ | OK | 4, 5 | M | 23 (N = 47) | 1 (N = 62), 1 PH | 0 | 14.1 | 15.81 |
| IS | IN | 1, 2 | F | 14 (N = 47) | 1 (N = 56), 1 PH | 0 | 15.4 | 18.26 |
| DB | DL | 1 | F | 11 (N = 24) | 1 (N = 38), 1 PH | 0 | 11.3 | 18.39 |
| KO | KL | 4, 5 | M | 22 (N = 31) | 4 (N = 42), 4 PH | 0 | 8.3 | 20.69 |
|  |  |  |  |  |  |  |  |  |
| *TOT* |  |  | F = 5 M = 8 | 198  (N = 402) | 51  (N = 549), 36 PH | 9 | 170.6 |  |

Age was calculated at the time of data collection. M: male; F: female. We report the number of head movements and vocal responses recorded with, in brackets, the total number of occurrences. ‘PH’ refers to the number of pant hoot calls among vocal responses.

Table S2 List of mature individuals considered in our study ordered by gregariousness

| *ID* | *Age* | *Sex* | *Head movement* | *Vocal responses* | *Spontaneous pant hoots* | *Focal duration (h)* | *Gregariousness* |
| --- | --- | --- | --- | --- | --- | --- | --- |
| KH | 11, 12 | F | 23 (N = 28) | 2 (N = 42) | 1 | 8.7 | 5.82 |
| ML | 44, 45 | F | 21 (N = 35) | 4 (N = 66) | 6 | 11.8 | 8.66 |
| KX | 13 | F | 12 (N = 20) | 2 (N = 36) | 3 | 9.9 | 9.11 |
| KW | 38 | F | 0 (N = 2) | 3 (N = 3) | 1 | 10 | 9.35 |
| HT | 41, 42 | F | 25 (N = 46) | 14 (N = 59) | 1 | 14.6 | 10.75 |
| KZ | 23 | M | 32 (N = 45) | 6 (N = 56) | 5 | 9.4 | 12.63 |
| SQ | 27 | M | 9 (N = 22) | 7 (N = 32) | 4 | 11.2 | 13.38 |
| EV | 15, 16 | F | 36 (N = 40) | 4 (N = 56) | 5 | 9.2 | 13.39 |
| OK | 23 | F | 32 (N = 54) | 15 (N =71) | 0 | 12.3 | 15.81 |
| ZD | 17, 18, 19 | M | 51 (N = 65) | 8 (N = 88) | 13 | 14.8 | 15.93 |
| KS | 15, 17 | M | 24 (N = 40) | 10 (N = 52) | 6 | 13.7 | 16.34 |
| IN | 20, 21 | F | 66 (N = 99) | 19 (N = 194) | 4 | 17.8 | 18.26 |
| DL | 17, 18 | F | 42 (N = 62) | 8 (N = 86) | 5 | 19.5 | 18.39 |
| ZL | 23, 24, 25 | M | 75 (N = 107) | 19 (N = 141) | 25 | 20.1 | 19.02 |
| JS | 12, 13, 14 | M | 58 (N = 98) | 12 (N = 123) | 17 | 27 | 19.11 |
| KT | 25, 26 | M | 79 (N = 127) | 25 (N = 146) | 38 | 34.6 | 19.71 |
| SM | 25, 26, 27 | M | 44 (N = 63) | 14 (N = 77) | 6 | 29 | 19.93 |
| KL | 40 | F | 8 (N = 20) | 1 (N = 28) | 0 | 13 | 20.69 |
| JN | 35 | F | 8 (N = 18) | 9 (N = 36) | 5 | 9.2 | 22.19 |
| NB | 57, 58 | F | 18 (N = 36) | 7 (N = 48) | 3 | 13.3 | 24.1 |
| MS | 26, 27, 28 | M | 78 (N = 114) | 22 (N = 147) | 31 | 33.9 | 24.34 |
| FK | 19, 20, 21 | M | 80 (N = 143) | 38 (N = 182) | 33 | 30 | 24.42 |
| PS | 20, 21, 22 | M | 47 (N = 67) | 8 (N = 95) | 19 | 25.5 | 25.86 |
| KC | 12, 13, 14 | M | 55 (N = 81) | 13 (N = 109) | 3 | 20.6 | 26.18 |
| HW | 25, 26 | M | 48 (N = 67) | 8 (N = 84) | 24 | 32.8 | 29.17 |
|  |  |  |  |  |  |  |  |
| *TOT* |  | F = 12 | 971 | 278 | 258 | 451.9 |  |
|  |  | M = 13 | (N = 1499) | (N = 2057) |  |  |  |

Age was calculated at the time of data collection. F: female; M: male. We report the number of head movements and vocal responses recorded with, in brackets, the total number of occurrences. All vocal responses were pant hoots.

*Study population*

Table S3 List of all individuals from the Sonso community

| *ID* | *Sex* | *Age category start* | *Age category end* | *Year of birth* |
| --- | --- | --- | --- | --- |
| AC | F | Infant | Infant | 2017 |
| AN | F | Adult | Adult | 1990 |
| BC | F | Adult | † | 1976 |
| BG | F | Sub-adult | * | 2004 |
| CD | F | Young adult | Young adult | 2003 |
| DL | F | Young adult | Young adult | 2002 |
| **DB** | **F** | **Infant** | **Infant** | **2018** |
| DR | F | Sub-adult | Young adult | 2004 |
| ER | F | Infant | Infant | 2019 |
| EV | F | Sub-adult | Young adult | 2004 |
| FA | F | Sub-adult | Sub-adult | 2006 |
| FH | F | Juvenile | Juvenile | 2013 |
| FL | F | Adult | Adult | 1979 |
| FK | M | Young adult | Adult | 1999 |
| **GF** | **M** | **Juvenile** | **Juvenile** | **2013** |
| GH | F | Infant | Infant | 2020 |
| GL | F | Adult | Adult | 1976 |
| GR | F | Sub-adult | * | 2006 |
| HD | M | Infant | Infant | 2017 |
| **HM** | **F** | **Juvenile** | **Juvenile** | **2013** |
| **HR** | **F** | **Juvenile** | **Sub-adult** | **2009** |
| HT | F | Adult | Adult | 1978 |
| HW | M | Adult | Adult | 1993 |
| IN | F | Young adult | Adult | 1999 |
| **IS** | **F** | **Infant** | **Infant** | **2017** |
| JA | F | Infant | Infant | 2018 |
| **JB** | **M** | **Juvenile** | **Juvenile** | **2011** |
| JL | F | Adult | Adult | 1990 |
| JN | F | Adult | Adult | 1984 |
| JS | M | Sub-adult | Sub-adult | 2006 |
| KA | F | Adult | Adult | 1998 |
| KB | F | Sub-adult | * | 2007 |
| KC | M | Sub-adult | Sub-adult | 2006 |
| KH | F | Sub-adult | Sub-adult | 2008 |
| KF | M | Infant | Juvenile | 2014 |
| KG | F | Adult | Adult | 1998 |
| **KJ** | **M** | **Juvenile** | **Juvenile** | **2013** |
| KL | F | Adult | Adult | 1979 |
| **KO** | **M** | **Infant** | **Juvenile** | **2014** |
| KP | F | Sub-adult | * | 2008 |
| **KQ** | **M** | **Infant** | **Infant** | **2016** |
| KS | M | Sub-adult | Adult | 2003 |
| KT | M | Adult | Adult | 1993 |
| KU | F | Adult | Adult | 1979 |
| KV | M | Infant | Juvenile | 2014 |
| KW | F | Adult | Adult | 1981 |
| KX | F | Sub-adult | Sub-adult | 2007 |
| KY | F | Adult | Adult | 1983 |
| KZ | M | Adult | † | 1995 |
| **MB** | **M** | **Juvenile** | **Sub-adult** | **2009** |
| MI | F | Sub-adult | Sub-adult | 2007 |
| MK | F | Adult | Adult | 1980 |
| ML | F | Adult | Adult | 1975 |
| MS | M | Adult | Adult | 1992 |
| **MZ** | **M** | **Infant** | **Infant** | **2015** |
| NB | F | Adult | Adult | 1962 |
| OK | F | Adult | Adult | 1996 |
| **OZ** | **M** | **Infant** | **Juvenile** | **2014** |
| PS | M | Young adult | Adult | 1998 |
| RF | F | Sub-adult | Sub-adult | 2007 |
| RH | F | Adult | Adult | 1965 |
| RM | F | Young adult | Young adult | 2002 |
| RN | M | Infant | † | 2018 |
| RS | F | Adult | Adult | 1997 |
| RY | M | Infant | † | 2013 |
| SM | M | Adult | Adult | 1993 |
| SQ | M | Adult | † | 1991 |
| ST | F | Sub-adult | * | 2007 |
| TJ | F | Adult | Adult | 1984 |
| **TW** | **F** | **Juvenile** | **Sub-adult** | **2010** |
| UP | F | Adult | Adult | 1999 |
| ZD | M | Young adult | Young adult | 2001 |
| ZF | M | Adult | † | 1982 |
| ZL | M | Adult | Adult | 1995 |

F: female; M: male. Study subjects are in bold.

† Subjects that died during the study period.

* (Potentially) emigrated females.

*Model structures*

Comparison with mature chimpanzee head movement response - final model:

Head movement ~ age category + sex + gregariousness + call from within party + solo call + number of males + number of females + activity + (1|Focal)

Comparison with mature chimpanzee vocal response - final model:

Vocal response ~ age category + sex + gregariousness + call from within party + solo call + number of males + number of females + activity + (1|Focal)

Head movement - final model:

Head movement ~ gregariousness + age + sex + mother chorus + call from within party + solo call + number of females + number of males + activity + (1|Focal)

Vocal response – final model:

Vocal response ~ sex*age + gregariousness*age + gregariousness*number of males + mother chorus + call from within party + solo call + number of females + activity + (1|Focal)

Maternal gregariousness and vocal exposure - final model:

Vocal exposure ~ age + gregariousness*sex

*Influential observations*

There were no outliers in the ‘head movement’ model (Cook’s distance: 0.947). The function detected 27 outliers in the ‘vocal response’ model, which belonged to eleven individuals (Cook’s distance: 1). To test their influence, we reran the same model in a leave one out fashion using a data set from which one study subject was excluded each time and repeated this for all 13 individuals. The difference between the full and null models was significant in all instances (S11 Table), supporting the idea that the ‘vocal response’ model used in our study fit the data well even when any of the study subjects were removed from the data. If a single individual was driving the significant effects from the ‘vocal response’ model, then we would observe only one model without the same effects after removing the data. Instead, we found that the removal of data from seven individuals (out of 13) did not produce the same model output and significant effects (S12 Table). More specifically, three models did not contain the same maternal gregariousness effects after removing the data, three models only contained the interaction between gregariousness and the control variable, and one model contained the same effects with an additional interaction with the sex of the focal (S12 Table). These results indicate that no individual was responsible by itself for the model output, including the effect of maternal gregariousness, of the ‘vocal response’ model. The complete model outputs from all 13 models are available for download at the following link: <https://osf.io/ze82d/?view_only=c58e1e88758a4a2bb7801c89991bc92b>

*Overfitting in the ‘vocal response’ model*

Because of the relatively small dataset and number of responses in the ‘vocal response’ model as well as the potential concerns regarding the confidence intervals, we conducted a series of additional tests to confirm the model was not overfitted and its explanatory power. A typical consequence of an overfitted model with poor power is a singular fit. We used the ‘isSingular’ function of the ‘lme4’ package (71) to determine whether any of the random effects’ covariance matrices were singular and found that they are not. Another characteristic of an overfitted model that is too complex is an R-squared value that is too high, which happens when the model describes the random error in the data instead of the relationship between the variables. We calculated the R-squared using the method developed by Nakagawa & Schielzeth (74) designed to work specifically on GLMM. We found that both marginal R-squared and conditional R-squared, which measure the variance explained by fixed factors, did not indicate the presence of an overfitted model (53% and 47% of the variance explained respectively). Finally, we performed cross-validation to test model overfitting. In order to assess how the model performed, we adopted the leave one out cross validation method, which is particularly suitable for small datasets and to test the influence of single datapoints. We removed a data point and constructed the model using the rest of the data points in the training set, repeated this procedure for each data point, and calculated a mean of all evaluations. We found that our model had an accuracy of 90.1% when making predictions on new data. Taken together, these additional tests indicate that the ‘vocal response’ model is not overfitted and has explanatory power.

Table S4 Summary of full vs. null model comparison test results

| ID removed | χ^2^ | *P* |
| --- | --- | --- |
| DB | 29.730 | <0.001 |
| GF | 33.153 | <0.001 |
| HM | 28.332 | <0.001 |
| HR | 31.227 | <0.001 |
| IS | 20.762 | <0.001 |
| JB | 28.972 | <0.001 |
| KJ | 20.376 | <0.001 |
| KO | 20.051 | <0.001 |
| KQ | 30.011 | <0.001 |
| MB | 27.904 | <0.001 |
| MZ | 31.081 | <0.001 |
| OZ | 28.511 | <0.001 |
| TW | 28.831 | <0.001 |

Table S5 Summary of comparisons between significant effects in the ‘vocal response’ model used in the study with each model where one individual was removed

| ID removed | Model’s significant effects | Same gregariousness effects? |
| --- | --- | --- |
| DB | mother chorus + sex*age + gregariousness*age + gregariousness*number of males | Yes |
| GF | mother chorus + activity + sex*age + gregariousness*age + gregariousness*number of males | Yes |
| HM | mother chorus + gregariousness*number of males | Only one of two. |
| HR | mother chorus + gregariousness*age + gregariousness*sex + gregariousness*number of males | Yes, plus another effect. |
| IS | mother chorus + number of females | No |
| JB | mother chorus + age + number of females + gregariousness*number of males | Only one of two. |
| KJ | mother chorus + number of females | No |
| KO | mother chorus + number of females | No |
| KQ | mother chorus + sex*age + gregariousness*age + gregariousness*number of males | Yes |
| MB | mother chorus + number of females + gregariousness*number of males | Only one of two. |
| MZ | mother chorus + number of females + sex*age + gregariousness*age + gregariousness*number of males | Yes |
| OZ | mother chorus + sex*age + gregariousness*age + gregariousness*number of males | Yes |
| TW | mother chorus + sex*age + gregariousness*age + gregariousness*number of males | Yes |

Supplementary Results

*Comparison with mature chimpanzee responses*


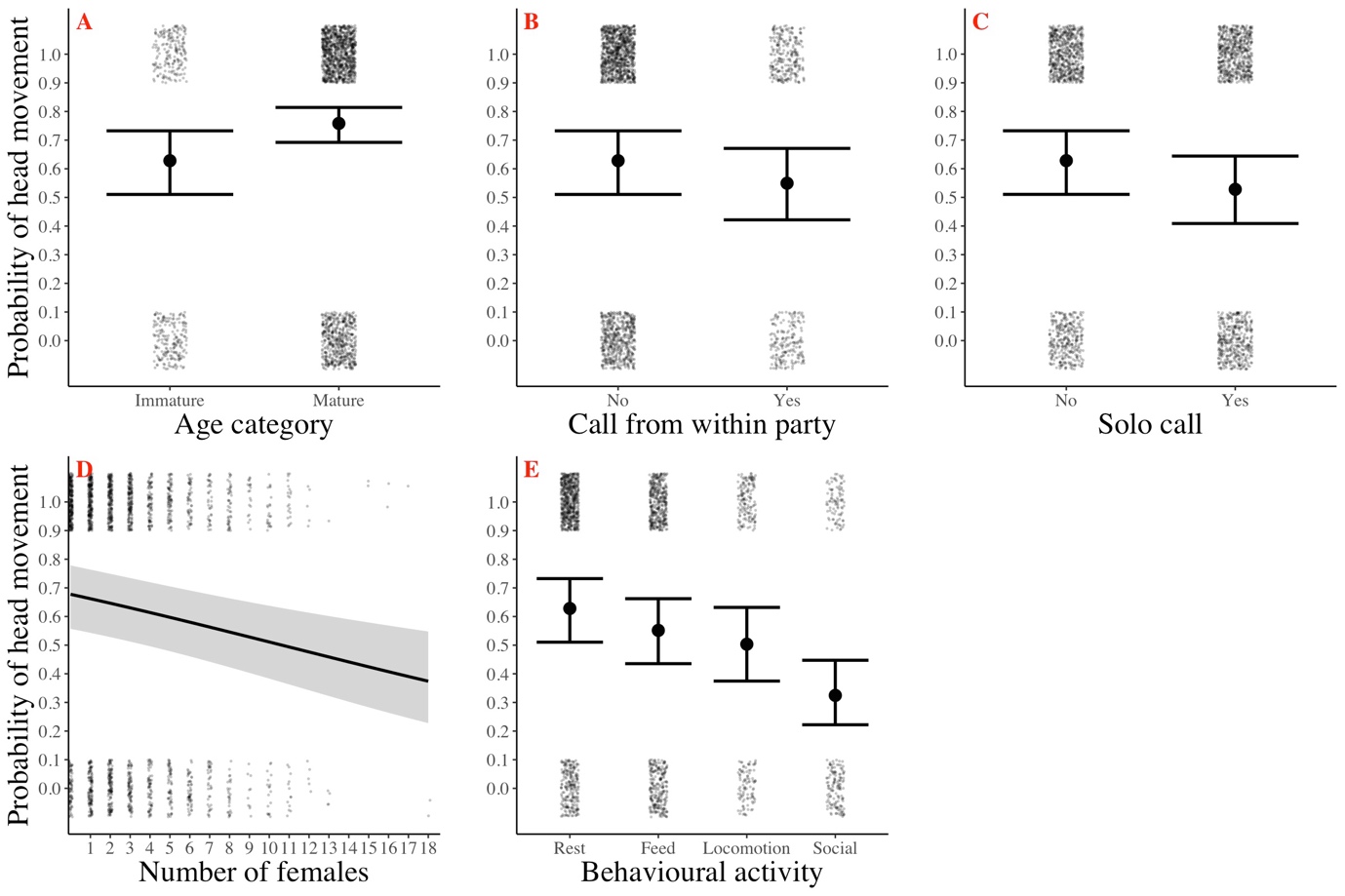


Figure S1 Likelihood of moving the head towards the source of the pant hoot depending on A) the age category of the focal (categorical), B) whether the call was from within the party (categorical), C) whether the call was a solo or group call (categorical), D) the number of females in the party (numerical), and E) the behavioural activity of the focal (categorical). Confidence bands and bars illustrate the standard errors (95%). Note that raw data, represented here with dots spread around the dependent variable values of either 0 or 1, do not express the influence of other factors included in the model.


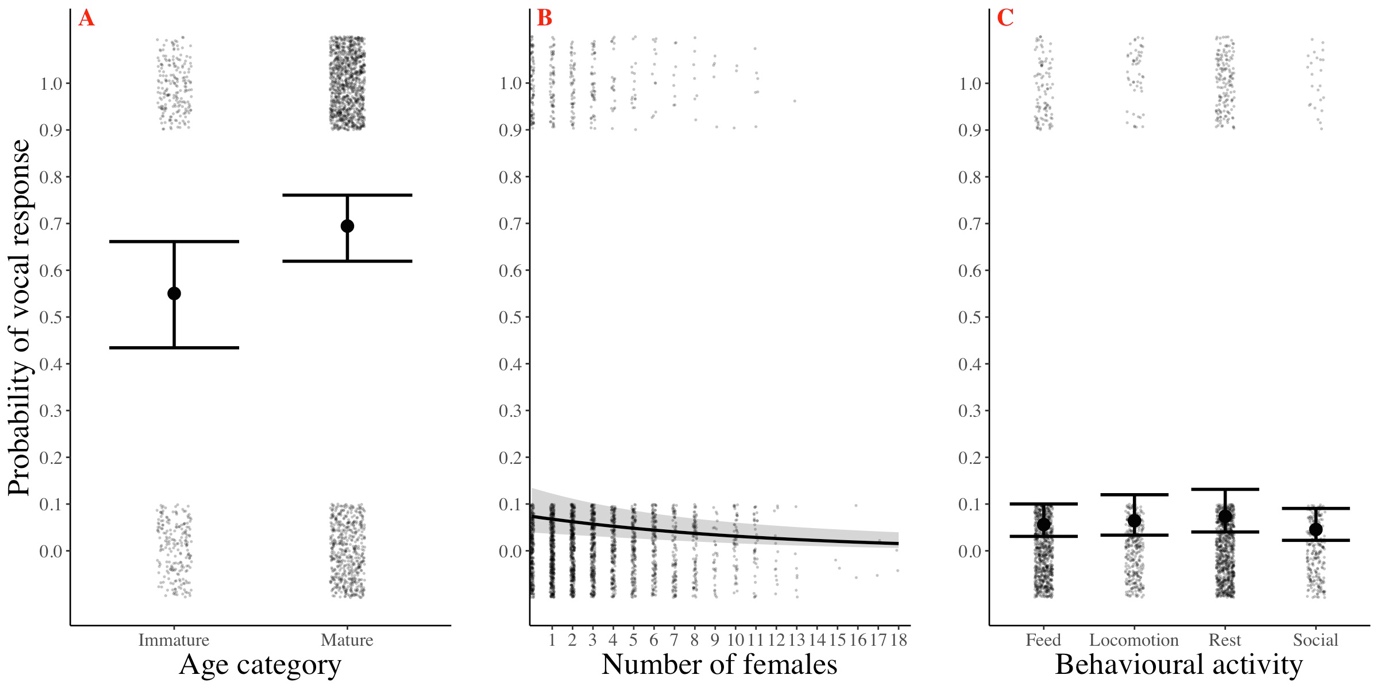


Figure S2 Likelihood of vocally responding to a pant hoot depending on A) the age category of the focal (categorical), B) the number of females in the party (numerical), and C) the behavioural activity of the focal (categorical). Confidence bands and bars illustrate the standard errors (95%). Note that raw data, represented here with dots spread around the dependent variable values of either 0 or 1, do not express the influence of other factors included in the model.

Table S6 Summary of random effects from the ‘head movement’ model

| Groups | Name | Variance | SD |
| --- | --- | --- | --- |
| Focal | (Intercept) | <0.001 | <0.001 |
| Number of observations: 401, groups: Focal (13) | | | |


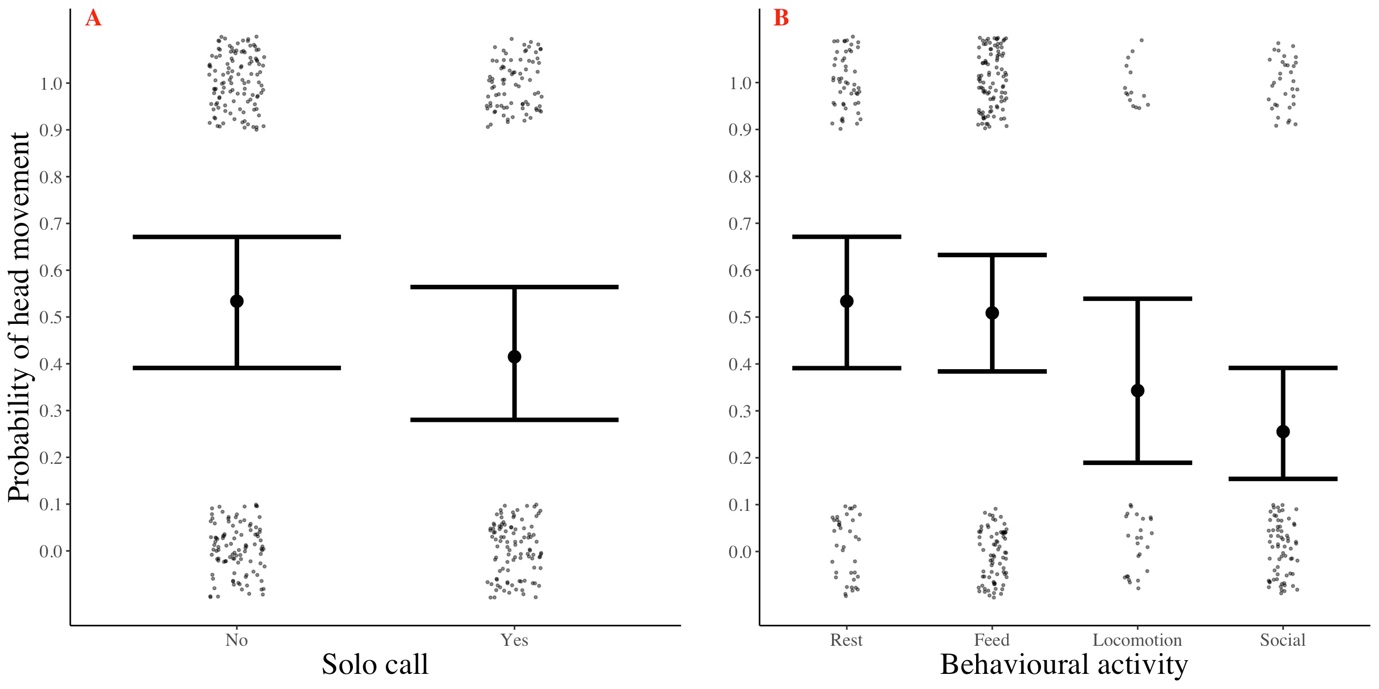


Figure S3 Likelihood of moving the head towards the source of the pant hoot depending on A) whether the call was a solo or group call (categorical), and B) the behavioural activity of the offspring (categorical). Bars illustrate the standard errors (95%). Note that raw data, represented here with dots spread around the dependent variable values of either 0 or 1, do not express the influence of other factors included in the model.

Table S7 Summary of random effects from the ‘vocal response’ model

| Groups | Name | Variance | SD |
| --- | --- | --- | --- |
| Focal | (Intercept) | 0.456 | 0.676 |
| Number of observations: 549, groups: Focal (13) | | | |
